# High-Efficiency Output Pressure Performance Using Capacitive Micromachined Ultrasonic Transducers with Substrate-Embedded Springs

**DOI:** 10.3390/s18082520

**Published:** 2018-08-02

**Authors:** Byung Chul Lee, Amin Nikoozadeh, Kwan Kyu Park, Butrus T. Khuri-Yakub

**Affiliations:** 1 Center for BioMicrosystems, Korea Institute of Science and Technology, Seoul 02792, Korea; 2 Department of Biomedical Engineering, University of Science and Technology, Daejeon 34113, Korea; 3 Department of Electrical Engineering, Stanford University, CA 94305, USA; amin.nikoozadeh@gmail.com; 4 Department of Mechanical Engineering, Hanyang University, Seoul 04763, Korea; kwankyu@hanyang.ac.kr

**Keywords:** capacitive micromachined ultrasonic transducers (CMUTs), substrate-embedded springs, nonflexural piston movement, high-efficiency output pressure

## Abstract

Capacitive micromachined ultrasonic transducers (CMUTs) with substrate-embedded springs offer highly efficient output pressure performance over conventional CMUTs, owing to their nonflexural parallel plate movement. The embedded silicon springs support thick Si piston plates, creating a large nonflexural average volume displacement efficiency in the operating frequency range from 1–3 MHz. Static and dynamic volume displacements of the nonflexural parallel plates were examined using white light interferometry and laser Doppler vibrometry. In addition, an output pressure measurement in immersion was performed using a hydrophone. The device showed a maximum transmission efficiency of 21 kPa/V, and an average volume displacement efficiency of 1.1 nm/V at 1.85 MHz with a low DC bias voltage of 55 V. The device element outperformed the lead zirconate titanate (PZT) ceramic HD3203, in the maximum transmission efficiency or the average volume displacement efficiency by 1.35 times. Furthermore, its average volume displacement efficiency reached almost 80% of the ideal state-of-the-art single-crystal relaxor ferroelectric materials PMN-0.33PT. Additionally, we confirmed that high-efficiency output pressure could be generated from the CMUT device, by quantitatively comparing the hydrophone measurement of a commercial PZT transducer.

## 1. Introduction

Capacitive micromachined ultrasonic transducers (CMUTs), are microelectromechanical system devices that make use of electrostatic actuation and detection of ultrasound, using multiple top moving plate geometries separated from the supportive substrate by vacuum gaps. Because CMUTs offer the advantages of improved fractional bandwidth (FBW), large operating frequency range, ease of fabrication from very small to large arrays with individual electrical connections, and simple integration with electronics, it has been proven through many demonstrations that this technology is one of the best candidates for advancing the state-of-the-art in medical ultrasound imaging [1]. Four-dimensional (4D) ultrasound imaging with a large number of two-dimensional (2D) CMUT array elements [2]; probe development of 2D CMUT array using row-column addressed electrodes [3,4]; compensated three-dimensional (3D) imaging techniques using column-row addressing architecture with 2D CMUT arrays [5,6]; volumetric intracardiac imaging with miniature one-dimensional (1D) and 2D ring arrays on catheters [7,8,9,10]; switchable high-intensity focused ultrasound (HIFU) therapy and 4D imaging using a single 2D CMUT array [11]; and photoacoustic imaging with 1D linear CMUT probes [12] are among the state-of-the-art demonstrations in medical applications.

**Figure 1:**
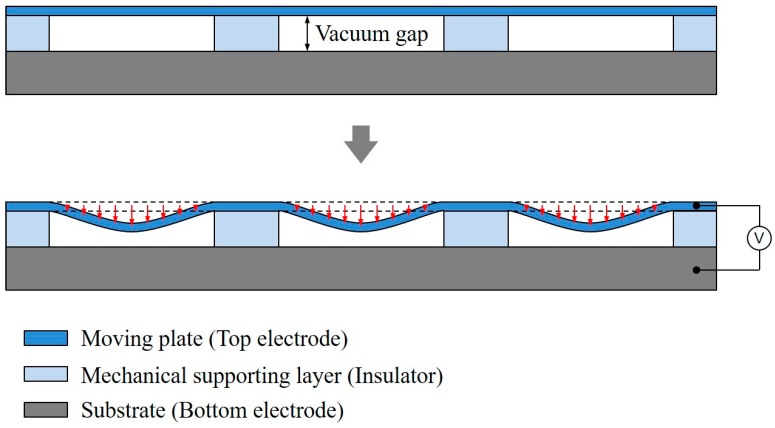
Cross-sectional schematic drawings of an element with 3 cells of a conventional capacitive micromachined ultrasonic transducers (CMUT). (**Top**) Initial state before applying DC and AC voltage, and (**Bottom**) flexural deformation of the moving plate due to electrostatic force.

Although the CMUT has received attention for the past two decades due to its versatility and advantages for medical applications, some basic improvements are necessary to further enhance its performance compared to piezoelectric transducers. Because of its inherent transduction mechanism, the transduction efficiencies (transmission efficiency and receiving sensitivity) and FBW, are so closely related that a performance trade-off is inevitable in a conventional CMUT design. For high transduction performance, the maximum average displacement of the CMUT plate has to be achieved with the given vacuum gap height. However, owing to its inherent flexural motion, as shown in Figure 1, the vacuum gap height in the CMUT structure is partially utilized for its transduction efficiency. Several solutions to these disadvantages, including CMUTs with piston-shaped membranes [13], dual-electrode CMUT with non-uniform membranes [14], indirectly clamped CMUTs [15], CMUTs with multiple moving membranes [16], and a collapse-mode CMUT with an embossed membrane [17] have been proposed.

In this paper, we introduce a CMUT with substrate-embedded springs which improve the transduction efficiency, particularly regarding output pressure performance at a lower voltage due to its piston-like motion. A theoretical comparison between the conventional CMUT and the proposed CMUT with substrate-embedded springs is provided, to demonstrate high efficiency on the transmit performance of the proposed ultrasonic transducer. Several micromachining techniques were utilized to build the CMUT with substrate-embedded springs. After fabricating the devices, we measured the electrical impedance using an impedance analyzer; and the acoustic transmit performances using a hydrophone and a laser Doppler vibrometer. We quantitatively compared the transmit performance of the proposed CMUT device with two PZT ceramic materials, considered one of the state-of-the-art single crystal piezoelectric materials, and a commercially available piezoelectric probe.

## 2. Working Principle

**Figure 2:**
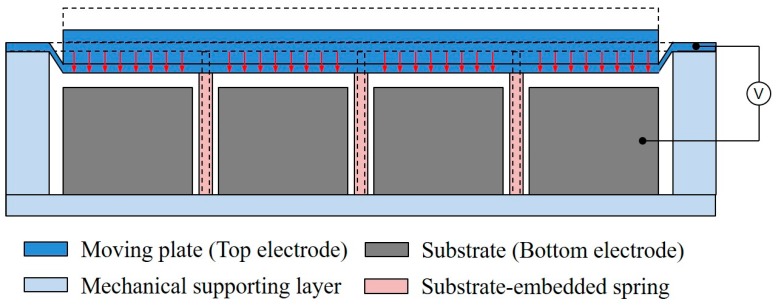
Cross-sectional schematic drawings of an element of CMUT with substrate-embedded springs. The dashed line represents the initial state of the CMUT with substrate-embedded springs before applying DC and AC voltage.

The CMUT with substrate-embedded springs has a compliant silicon post or rod structure, supporting the rigid thick moving plate or piston top plate, as shown in Figure 2. As compared to the operation principle of the conventional CMUT, the compliant silicon post or rod structure provides the spring a constant in the mass-spring-damper system. Since the compression and restoration of the plate movement are not provided by the plate itself, but from the separate silicon springs, the CMUT with substrate-embedded springs resembles an ideal electrostatic piston with parallel movement.

To compare the average displacement between the conventional CMUT and the CMUT with substrate-embedded springs, the static average displacement of a conventional circular CMUT was calculated using Kirchhoff’s thin plate bending theory [18] as

(1) $$\frac{P}{D}=\nabla^{2}\nabla^{2}u\;where\;D=\frac{2h^{3}E}{3\left(1-v^{2}\right)}$$

(2) $$u=\frac{P_{0}b}{8a^{2}D}\{\begin{array}{c} \left(a^{2}-b^{2}\right)\left(a^{2}+r^{2}\right)+2a^{2}\left(b^{2}+r^{2}\right)\ln\left(\frac{b}{a}\right)\;r\leq b\\ \left(a^{2}+b^{2}\right)\left(a^{2}-r^{2}\right)+2a^{2}\left(b^{2}+r^{2}\right)\ln\left(\frac{r}{a}\right)\;r>b \end{array}$$

(3) $$u_{avg}=\;\frac{1}{A_{tot}}\int_{A_{tot}}udA$$

where *P*_0_ is the uniform line external pressure distributed along a circle with radius *b*, *a* is a typical radius of the whole plate, *u* is the corresponding displacement, *D* is the flexural rigidity of the plate, *h* is the plate thickness, *E* is the plate material’s Young’s modulus, *v* is the plate material’s Poisson’s ratio, and *A_tot_* is the total plate area. We assumed that the plate had no internal tensile or compressive stress so that the Kirchhoff’s equation could be expressed merely as Equation (1). From the modified Bessel functions and the assumption of the negligible internal stress in the plate, the solutions for the circular CMUT can be greatly simplified as Equation (2). With the segmentation of the plate and superposition of the displacement [19], the calculation result of *u_avg_* showed that the CMUT plate with substrate-embedded springs can displace on average almost three times, i.e., 2.78 at 80% of the pull-in voltage and 2.85 at 99% of the pull-in voltage, more than the conventional CMUT plate. Thus, it can be surmised that the CMUT with substrate-embedded springs transmits ultrasound wave more efficiently, promising improvements in performance by achieving a higher average displacement for a given electric field. The output pressure *P* is proportional to the average displacement based on the acoustic wave theory [20]:

(4) $$P=Re\left(Z_{m}V\right)=Re(Z_{m})\times \omega \times \bar{x}$$

where *Z_m_* is the mechanical impedance of the medium, *V* is the velocity of the acoustic wave particle, *ω* is the operating frequency, and $\bar{x}$ is the average displacement amplitude of the acoustic wave particle, which is equivalent to the plate displacement.

## 3. Design and Fabrication

For the design of the CMUT structure with substrate-embedded springs, we previously simulated and investigated the finite element analysis (FEA) model with a commercial simulator (ANSYS, Inc., Canonsburg, PA, USA) [21]. Based on the simulation results, four 100 μm × 100 μm silicon piston top plates with thicknesses of 20 μm were used to compose a 2D CMUT element, with an operating frequency around 2 MHz in immersion. Each piston top plate was attached to the substrate with nine silicon post springs, where each spring was 4 μm in diameter and 50 μm in length. To investigate the independence between the spring and the mass components in the piston top plate, the top plates were patterned as two different types; one has a plane feature and the other has a truss structure.

Wafer bonding of two silicon-on-insulator wafers that have undergone a deep reactive ion etching process, and a chemical mechanical polishing process, provides good dimensional control in fabricating the structure. The detailed fabrication process was similar to the previous work [22]. The bottom side starting substrate was a silicon-on-insulator (SOI) wafer having a device layer of 50 μm, 2 μm thick BOX layer, and 350 μm thick handle layer from Ultrasil (Hayward, CA, USA). The crystal orientation of the SOI device layer was set up to be (100). To provide the bottom electrode, the electrical resistivity of the device layer was chosen as 0.003 Ω cm. The top side 8-inch SOI substrate had a device layer of 200 nm, 150 nm thick BOX layer, and 725 μm thick handle layer from Soitec (Grenoble, France). To increase the conductivity of the device layer which is served as the top electrode, we epitaxially grew 50 nm highly doped silicon layer on the 200 nm thick device layer. The 3 × 10^19^ cm^−3^ phosphorus was added during the epitaxial growth from LSQI (Reno, NV, USA). Our facility provided most of the process in 4-inch bases, so that the 8-inch SOI wafer was cut into two 4-inch wafers from Ultrasil (Hayward, CA, USA).

**Figure 3:**
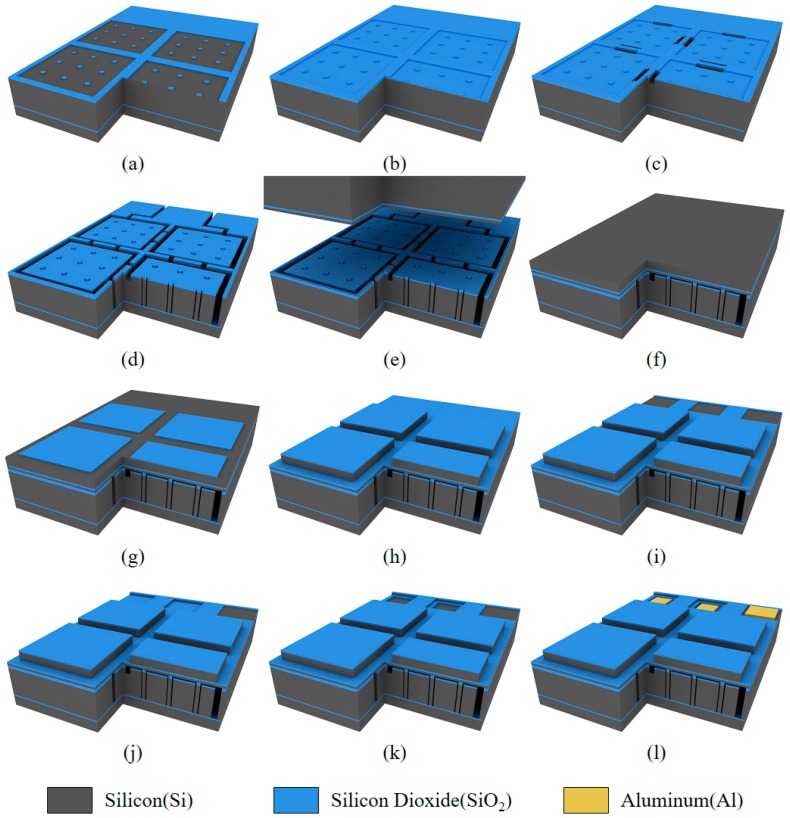
The fabrication process flow of the CMUT with substrate-embedded springs. (**a**) 250 nm silicon dioxide patterning for vacuum gap area; (**b**) Thermal oxidation for defining 150 nm vacuum gap height; (**c**) Formation of electrical connection bridges using RIE; (**d**) DRIE process step for defining four CMUT cells and 36 silicon spring posts; (**e**) Direct wafer fusion bonding of the processed bottom SOI wafer and a top blank SOI wafer; (**f**) Wafer grinding and polishing for piston top plate; (**g**) Thermal oxidation and patterning for top plate mask; (**h**) DRIE step for piston top plates; (**i**) BOX layer opening for electrical contacts; (**j**) Silicon device layer etching for bottom electrode access; (**k**) BOX layer etching for bottom electrodes; (**l**) Metal deposition and patterning for three electrode pads.

First, to accurately control the vacuum gap height, double oxidation with two steps of 200 nm oxidation after 250 nm oxidation led to the vacuum gap height of 150 nm (Figure 3a,b). The partially etched silicon block was formed to connect all the four cells into one element using RIE process (Figure 3c). The DRIE process defined the 2D CMUT element and the lateral dimensions of the silicon post springs (Figure 3d). The donut shape mask was used, having a trench opening width of 3 μm. In this case, the pitch of the nine posts was set to be 30 μm. The top side SOI wafer was roughly aligned with the bare eye on top of the bottom processed wafer. Before placing the top SOI wafer, RCA1 cleaning process was conducted to provide chemical activation of the two bonding surfaces. The wafer bonding process was done under 600 N of a bonding force with the chamber pressure of 4 × 10^−5^ mbar (Figure 3e). We put the bonded wafer for 9 h in the annealing furnace, with the condition of nitrogen ambient and 1100 °C. Subsequently, the handle layer of the top wafer was ground and polished, until the plate thickness reached 20 μm (Figure 3f). To accurately define the truss structure, 100 nm of silicon dioxide mask was grown and patterned (Figure 3g). Sequentially, the top plate was defined using DRIE (Figure 3g), and all the layers, such as 150 nm BOX layer, 250 nm device layer, and 250 nm silicon dioxide layer, were consecutively etched to make electrical access to the bottom electrodes (Figure 3i–k). Finally, 500 nm aluminum was deposited and patterned, as shown in Figure 3l.

**Figure 4:**
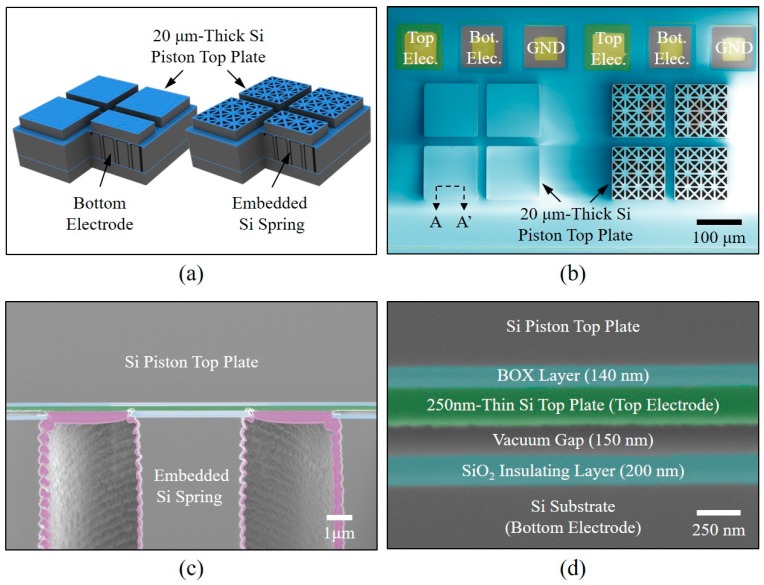
(**a**) A perspective schematic drawing of two different 2D CMUT elements with substrate-embedded springs. (**b**) A top-view colored SEM image of the two different 2D CMUT elements. (**c**) A cross-sectional SEM image of an embedded Si spring bonded with a Si piston top plate (the cross-sectional line is shown in Figure 3b). (**d**) A magnified cross-sectional SEM image of the vacuum gap area.

Two different types of the fabricated 2D CMUT elements, the plane type and the truss type, is illustrated in Figure 4a. Figure 4b, shows the top-view colored field emission scanning electron microscopy (SEM) images of the two different 2D CMUT elements. The mass of the truss structure was defined using the photolithographic process and was 84.6% of the mass of the plane structure. Focused ion beam (FIB) milling revealed that the embedded silicon post spring was well-bonded to the piston top plate, as shown in Figure 4c. In the picture, the pink colored area represents silicon debris redeposited during the milling process. Figure 4d, shows a magnified SEM picture of the vacuum gap area. The vacuum gap was 150 ± 2 nm, the thickness of the silicon top plate as the top electrode was 250 ± 5 nm, and the thickness of the silicon dioxide insulating layer was 200 ± 3 nm. This confirmed our tight vertical dimension control from the fabrication process.

## 4. Experimental Results

**Figure 5:**
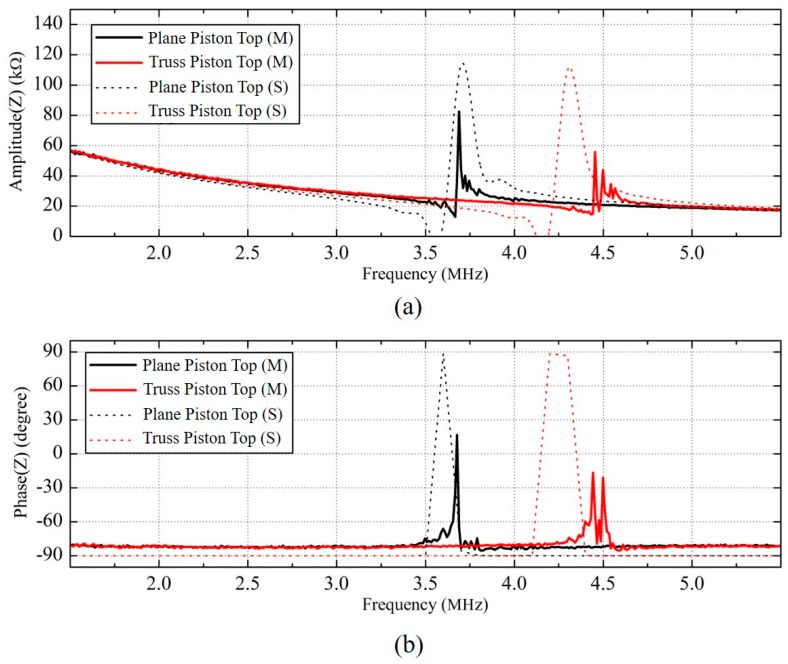
Electrical input impedance measurement results of the plane and truss 2D CMUTs biased at 40 V DC in air: (**a**) the amplitude part and (**b**) the phase part. The dotted line represented the ANSYS simulation results of each type of the CMUTs.

The amplitude (Figure 5a) and phase (Figure 5b) parts of the electrical input impedance of the plane and truss 2D CMUT with substrate-embedded springs, were measured using an impedance analyzer (Agilent 4294A, Santa Clara, CA, USA). The DC bias from the high voltage power supply (SRS PS310, Stanford Research Systems, Stanford, CA, USA) was applied to the 2D CMUT through the DC arm of the bias T circuit. A small signal AC voltage of 100 mV, was superimposed through the bias T circuit. After verifying the pull-in voltage of 78 V, the bias voltage was set to be 40 V. Because the mass of the truss 2D CMUT was 84.6% of the mass of the plane structure, the resonant frequency of the truss 2D CMUT showed higher resonant and anti-resonant peaks, in Figure 5. We also verified that the resonant frequencies from ANSYS, deviated less than 6% from the impedance measurement results.

**Figure 6:**
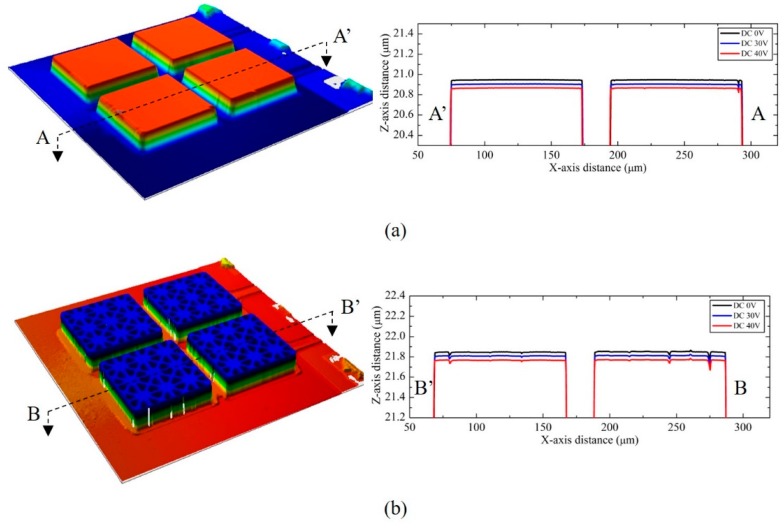
(**a**) A 3D surface profile of the plane 2D CMUT element at 0 V DC bias, and its corresponding 2D profile plot along the line A−A’ at different DC biases, 0 V (black), 30 V (blue), and 40 V (red), respectively. (**b**) A 3D surface profile of the truss 2D CMUT element at 0 V DC bias, and its corresponding 2D profile plot along the line B−B’ at different DC biases, 0 V (black), 30 V (blue), and 40 V (red), respectively.

A high-resolution white light interferometer (CCI HD, Taylor Hobson, Inc., Leicester, UK) was used to characterize the static displacement of the CMUT elements with substrate-embedded springs, as a function of the DC biases (Figure 6). The static deflection of each top plate with no DC bias voltage, was measured to set the baseline for the static displacement. This ensured no significant flexural bending of the top plate for each plate type from atmospheric pressure, as compared to the typical CMUT, which showed the apparent flexural bending feature at the center of its plate, by the pressure difference between the vacuum cavity and the atmosphere. As shown in the 2D profile plots in Figure 6, the nonflexural parallel plate displacement is demonstrated for various DC biases. Since the number of silicon post springs was the same between the plane and truss 2D CMUTs, the pull-in voltage of the devices was apparently measured to be the same value of 78 V, and the same amount of static displacement for the same DC bias was observed.

**Figure 7:**
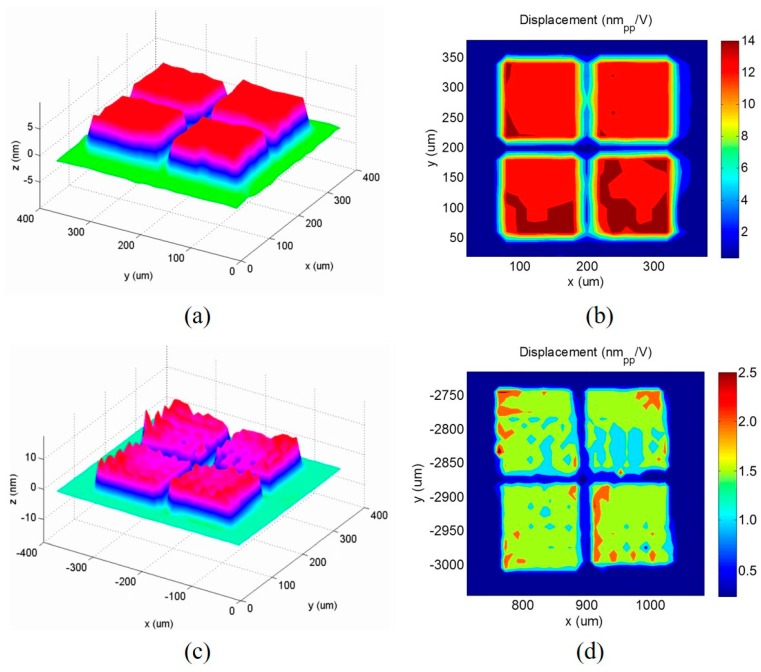
The dynamic plate displacement measurements from a laser Doppler vibrometer. (**a**) A 3D perspective view at the moment of the positive maximum peak displacement of the plane 2D CMUT element in air and (**b**) its contour plot of the maximum peak-to-peak displacement over the measurement time. (**c**) A 3D perspective view at the moment of the positive maximum peak displacement of the truss 2D CMUT element in oil and (**d**) its contour plot of the maximum peak-to-peak displacement over the measurement time.

The dynamic response of the 2D CMUT elements with substrate-embedded springs, was measured using a laser Doppler vibrometer (OFV-511, Polytec GmbH, Waldbronn, Germany) with in-house custom-built microscopy. The laser focused from the lens was scanned for 40 steps with a step size of 10 μm, along the X and Y axes. An AC function generator (Agilent 33250A, Agilent, Inc., Santa Clara, CA, USA), was connected to an in-house custom-built interface printed circuit board for applying the bias voltage and the AC signal to the CMUT devices. With a DC bias voltage of 40 V superposed onto a continuous wave pulse of 1 V and 3.5 MHz, Figure 7a explicitly demonstrates the nonflexural parallel piston movement of the plane top plate in air. In addition, the measurement verifies that all four piston top plates were in-phase during the transduction. The maximum peak-to-peak displacement efficiency in air was 12 nm/V, which was almost the same as the maximum average displacement efficiency (Figure 7b). The measurement of the dynamic displacement was also examined in soybean oil using the truss element. The CMUT device was excited using a unipolar rectangular pulse of 20 V and 2 MHz. The device was initially biased with a 35 V DC voltage. Figure 7c shows a three-dimensional (3D) perspective view of the truss piston plate displacement at the maximum positive peak. The maximum peak-to-peak displacement efficiency in oil, was calculated to be 1.1 nm/V, as shown in Figure 7d.

**Figure 8:**
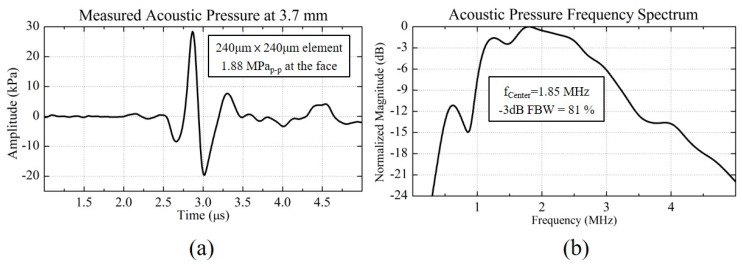
Measured acoustic pressure of the plane 2D CMUT element at 3.7 mm distance. (**a**) Transient pressure and (**b**) its frequency spectrum.

A hydrophone measurement was carried out in immersion to obtain the maximum output pressure and the transmission efficiency. A hydrophone (HGL-0200, Onda, Co., Sunnyvale, CA, USA), linked with a pre-amplifier was connected to a digitizing oscilloscope (Infiniium 54825A, Agilent, Inc., Santa Clara, CA, USA), and immersed in oil to monitor the pressure field from the 2D CMUT elements. The plane 2D CMUT element was biased at 55 V, which was about 70% of the pull-in voltage. A 2 MHz, 90 V peak-to-peak single sine pulse was superposed onto the DC bias voltage. The plane 2D CMUT, operated in the normal mode but not in the collapse mode because, the single sine pulse did not make the system statically in-phase and transiently drove the CMUT; even though the arithmetic sum of the applied voltage exceeded the pull-in DC voltage [23]. The distance between the hydrophone and the 2D CMUT element was set to be 3.7 mm, which was sufficient to detect the far-field signal after the focal zone. Figure 8 shows the transient pressure amplitude and its frequency spectrum for the plane 2D CMUT element. The output pressure at the surface of the 2D CMUT element, was calculated considering the attenuation and diffraction loss in the medium [24]. The corresponding maximum transient pressure at the surface was 1.88 MPa peak-to-peak, with a transmission efficiency of 21 kPa/V. Using Equation (3), the maximum average displacement efficiency was calculated to be 1.13 nm/V. The calculated maximum average displacement efficiency was closely matched to the measurement from the laser Doppler vibrometer. As shown in Figure 8b, a −3 dB FBW of 81% around the center frequency of 1.85 MHz was obtained.

## 5. Transmit Efficiency Comparison of PZT Ceramic vs. CMUT with Substrate-Embedded Springs

We compared the measurement data of our device with FEA simulation data of a lead zirconate titanate (PZT) ceramic HD3203 (CTS Corp., Lisle, IL, USA), a PZT ceramic PZ21 (Meggitt A/S, Kvistgaard, Denmark), and a single crystal PMN-33%PT (0.67Pb(Mg_0.33_Nb_0.67_)O_3_-0.33PbTiO_3_) from literature in Reference [25]. We also measured the pressure field, of a commercial PZT probe (model SP1-5, Alpinion Medical Systems, Co., Ltd., Anyang, Korea). Table 1 summarizes the hydrophone measurement results of the 1D phased array PZT probe. Table 2 summarizes the transmission performance comparison between the PZT ceramic HD3203; the PZT ceramic PZ21; the single crystal PMN-0.33PT; the measured commercial PZT probe; and the 2D CMUT element with substrate-embedded springs. Although single crystal piezoelectric materials, such as lead magnesium niobate-lead titanate (PMN-PT), and lead zinc niobate-lead titanate (PZN-PT) generally offer the highest transmission efficiency, the 2D CMUT element outperformed the two PZT ceramics HD3203 and PZ21, and the commercial 1D phased array probe in the maximum output pressure efficiency or the average volume displacement efficiency by 1.35 times, 1.40 times, and 1.59 times. The measured average volume displacement efficiency of the CMUT reached almost 80% of the simulated value of the PMN-0.33PT. In the FEA data of the two PZT ceramic materials and the single crystal PMN-0.33PT, the simulation was performed with the ideal backing layer model which was assumed to be infinitely thick, while the hydrophone measurement results showed that all the acoustic attenuation and loss were considered. Thus, taking this possible loss of the ideal PMN-0.33PT performance into account, the 2D CMUT element can provide an almost comparable average volume displacement efficiency of the state-of-the-art single-crystal piezoelectric materials. Also, since lead-containing materials possibly cause harmful effects on the environment and public health [26,27], the comparison result showed that the 2D CMUT element with the comparable transmission performance of the PZT ceramics, could be one of the alternatives to the lead-based piezoelectric materials.

## 6. Conclusions

We have shown the concept, fabrication, and characterization of CMUT elements with substrate-embedded springs achieving high output pressure efficiency. The nonflexural parallel piston plate movement of the device, by the embedded springs induces high average volume displacement efficiency with low DC and AC voltage. With its high efficiency and low voltage operation, we believe the CMUT with substrate-embedded springs can be significantly applied to medical applications such as HIFU, ultrasound elastography, point-of-care, and portable/wearable devices. Since the CMUT has the advantage of being fabricated in 2D arrays and integrated with integrated circuits, we plan to fabricate 1D CMUT arrays and systemize them for acoustic radiation force impulse imaging.

## Figures and Tables

**Table 1:** Hydrophone measurement result of the 1D phased array lead zirconate titanate (PZT) probe.

TX Frequency (MHz)	V_pp_	Pressure @ Focal Spot (Mpa)	Element Number	Focus (mm)	Acoustic Gain	Attenuation (dB/MHz/mm)
2.00	30	0.66	64	52	2.21	0.75
2.00	45	1.04	64	52	2.21	0.75
2.00	60	1.33	64	52	2.21	0.75

**Table 2:** Transmission performance comparison between PZT ceramics 3203HD, PZ21 and the CMUT with substrate-embedded springs.

	PZT Ceramic (3203HD) ^1^	PZT Ceramic (PZ21) ^1^	PZT Ceramic (PMN-0.33PT) ^1^	A Commercial PZT Probe ^2^	CMUT with Substrate-Embedded Springs
Frequency (MHz)	1.85	1.85	1.85	2.00	1.85
Max. Pressure (kPa)	1431 @ 90 V_pp_	924 @ 60 V_pp_	2457 @ 90 V_pp_	802 @ 60 V_pp_	1880 ^3^ @ 90 V_pp_
Transmit Pressure Efficiency (kPa/V)	15.9	15.4	27.3	13.4	21.5
Average Volume Displacement Efficiency ^4^ (nm/V)	0.804	0.779	1.43	0.711	1.13

^1^ The simulation and calculation data from Reference [25]. ^2^ The maximum pressure value at the surface of the transducer was calculated to consider the acoustic attenuation and acoustic gain from the acoustic lens. ^3^ The maximum pressure at the surface of the transducer was calculated to consider the acoustic attenuation and diffraction loss. ^4^ This value was calculated using Equation (4).
